# *Neobenedenia melleni* Parasite of Red Snapper,* Lutjanus erythropterus*, with Regression Statistical Analysis between Fish Length, Temperature, and Parasitic Intensity in Infected Fish, Cultured at Jerejak Island, Penang, Malaysia

**DOI:** 10.1155/2016/1946283

**Published:** 2016-04-06

**Authors:** Rajiv Ravi, Zary Shariman Yahaya

**Affiliations:** School of Biological Sciences, Universiti Sains Malaysia, Minden, 11800 Penang, Malaysia

## Abstract

The fish parasites collected from* Lutjanus erythropterus* fish species showed a correlation with parasitic intensity, fish size, and temperature, and statistical model summary was produced using SPSS version 20, statistical software. Statistical model summary concluded that among the variables which significantly predict the prevalence of* Neobenedenia melleni* parasites are fish length and water temperature, both significant at 1% and 5%. Furthermore, the increase in one unit of fish length, holding other variables constant, increases the prevalence of parasite by approximately 1 (0.7≈1) unit. Also, increasing the temperature from 32°C to 33°C will positively increase the number of parasites by approximately 0.32 units, holding other variables constant. The model can be summarized as estimated number of* Neobenedenia melleni* parasites = 8.2 + 0.7 ⁎ (fish length) + 0.32 ⁎ (water temperature). Next, this study has also shown the DNA sequence and parasitic morphology of* Neobenedenia melleni*. Nucleotide sequence for 18s ribosomal gene RNA in this study showed 99% similarity with* N. melleni *EU707804.1 from GenBank. Finally, all the sequence of* Neobenedenia melleni* in this study was deposited in GenBank with accession numbers of KU843501, KU843502, KU843503, and KU843504.

## 1. Introduction

Information and quantitative data on cultured fishes are limited in Southeast Asia. However, the existing data explains closely that similar species of fishes are cultured throughout the Southeast Asian region and the dominant parasites found infecting each species of these cultured marine fishes are similar [1–5]. Numerous studies on the parasitic fauna of marine fishes have indicated that the dominant parasites in each fish species are the same regardless of the wild or cultured [3, 6]. The main difference between the wild and cultured, diseased marine fishes is that the number and variety of parasites in both groups of cultured fishes greatly exceed those found in the wild fishes [7].

Monogenean parasites have been recognized as serious pathogens of fish in sea cage aquaculture [8–10]. Monogenea parasites have no intermediate host, predominantly parasitise the external surfaces of fish, and display two distinctive diets that traditionally divide them into two subclasses, the blood feeding polyopisthocotylea and the epithelial feeding monopisthocotylea [11]. These are sometimes named Heteronchoinea and Polyonchoinea, respectively [12]. These subclasses are united by various morphological synapomorphic larvae with three ciliated zones, adults, and larvae with two pairs of pigmented eyes, one pair of ventral anchors (hamuli), and one egg filament [13]. Inference about the Monogenea parasite is monophyletic, which has been ubiquitous for decades [13–18].* Neobenedenia melleni* (MacCallum, 1927) Yamaguti, 1963, a capsalid monogenean of the subfamily* Benedenia* sp., is disreputable as a widespread pathogen of many teleost species in aquaculture [19]. This parasite feeds on epithelial cells mucus of host fish, which gives increased effects towards irritation and mucus hyperproduction of their hosts [20]. Like most of other monogenean groups,* benedenids* have traditionally been identified to species on the basis of morphological characters such as the shape of posterior hamuli, the type of anterior attachment organ, and the length of uterus, vitelline reservoir, and the type and relative size of testes [21]. Though it has been argued for a long time that morphological characters based identification of parasite can be affected, to a large extent, by extrinsic factors such as the age of parasite, environmental temperature, and even artifacts caused by various dealings for specimen processing, as discussed by Li et al. [22], most monogeneans could be appropriately distinguished because of their high level of host specification. However,* Neobenedenia melleni* does not obey the rule because it has been reported from more than 100 teleost fish species belonging to more than 30 families with worldwide distributions [23].

To date, there is no reference yet that has been done on the correlations of* Neobenedenia melleni* parasite infestations to the fish size, temperature, and salinity factors in Malaysia. This is an important aspect of research as it will benefit fish farmers for aquaculture industry to predict any fish parasite infestation in their farm and to take initiatives to prevent parasitic infections. Thus, the objective of this study is to show the prevalence and statistical analysis of* Neobenedenia melleni* parasite to the fish size and water temperature in* Lutjanus erythropterus* fish species sampled from cage culture Jerejak Island, Penang, Peninsular Malaysia. Furthermore, we have successfully identified the parasite species using morphology and molecular approach.

## 2. Materials and Methods

### 2.1. Sampling Locality and Parasite Collection

The experiment was carried out with 400 fish specimens of cultured* Lutjanus erythropterus* fish species from Jerejak Island, Penang, Peninsular Malaysia (5.320097 longitude, 100.3189185 latitude). The length (cm) of each fish was measured prior to parasite examination. Fresh water medium was used as anesthetics to reduce the stress as well as for easy handling. After the fish has been anaesthetized, presence of ectoparasite was examined via external fish body examination and direct observation under light microscope [24]. The site specificity of parasite was obtained from head, body, and both sides of inner operculum.

First morphological identification of parasite was done by first staining the parasite with a few drops of lactophenol solutions (200 mL lactic acid, 200 g/L phenol, 400 mL glycerol, and 200 mL deionized water). Upon staining, slides were observed under the compound microscope (Leica, USA). Parasite found was taken out carefully from the infected area, and then the number of parasites obtained from each fish was recorded, preserved with 70% ethanol solution in universal bottle for further examination. After the pictures of parasites had been taken, identification of parasites collected was done by morphological observation using identification keys as suggested by Kua et al. [25, 26].

### 2.2. Morphological Method Using Scanning Electron Microscope

Second morphological identification was done using the Supra 50vp ultra high resolution LEO analytical Fesem, scanning electron microscope. Electron microscopic sample preparation was done as suggested by protocol of Supra 50vp ultra high resolution LEO analytical Fesem, scanning electron microscope guide manual. Firstly, suspended samples in ethanol were put into serial dilution of 90%, 80%, and 70% ethanol. Then, a droplet of the suspension was placed on a carbon film coated 400-mesh copper grid for 1–3 minutes. The droplet is then dried using pieces of filter paper. The grid was then placed in a filter paper lined Petri dish for preservation in desiccator. Finally, imaging would be carried out after 3 days of preservation.

### 2.3. Molecular Method Using DNA Identification

The genomic DNA extraction and purification of the parasite was performed using the procedures provided by Qiagen DNeasy Blood and Tissue Kit (Qiagen, Inc., Valencia, CA, USA). Purified genomic DNA was eluted by adding 100 *μ*L of buffer AE to the same spin column in a new Eppendorf tube and centrifuged at 5200 g for 1 min. The centrifuge step was repeated again for a total of 200 *μ*L sample volume. DNA sample was stored at −20°C and concentration measured with ACT-Gene NanoDrop spectrophotometer (ASP 2680, Taiwan).

Ribosomal RNA 18s partial sequences were amplified from purified genomic DNA using the specific primers 18sF (5′-GCG CGA GAG GTG AAA TTC AT-3′) as forward primer and 18sR (5′-AGT TTA CCC AGC CCT TTC GA-3′) as reverse primer, as discussed by Dang et al. [27] synthesized by MyTACG Bioscience (Malaysia). Polymerase chain reaction (PCR) was carried out using a total volume of 25 *μ*L master mix solutions (14 *μ*L of ddH_2_O, 2.5 *μ*L of Promega PCR buffer, 3 *μ*L of Promega MgCl_2_ solutions, 1 *μ*L of Promega dNTP, 1 *μ*L of each forward primer and reverse primer, 2 *μ*L of DNA template, and 0.5 *μ*L of Promega Go Taq DNA polymerase). Standard cycle conditions for PCR were set accordingly by initial denaturation for 10 min at 95°C, followed by 35 cycles of 30 s at 95°C, 30 s at 50°C, 60 s at 72°C, and final elongation of 7 minutes at 72°C. The whole PCR was carried out in MyCycler thermal cycler Bio-Rad PCR systems (USA). Purification of PCR product was performed using the procedure and materials provided in a QIAquick PCR Purification Kit (Qiagen, Inc.). Amplification products were sequenced in both directions by MyTACG Bioscience Company (Malaysia).

### 2.4. DNA Alignment and Phylogenetic Analysis

Alignment analysis of nucleic acid sequences was performed using ClustalW2 MEGA 5. Distance-based tree approach to species identification was conducted using MEGA 5 software. A BLAST search was conducted with DNA sequence that was amplified. Using MEGA 5, as discussed by Tamura et al. [28], a distance-based tree approach to species identification was carried out by neighbour-joining the 18s sequences of recorded species from the BLAST search and those analyzed in this study. Pairwise distance calculation is done using MEGA 5 analysis tools and the Kimura 2-parameter [29]; method serves as the substitution model. In addition, the bootstrap method was deployed as test of phylogeny using 1000 bootstrap replications. Finally, all the sequences were submitted in GenBank according to submission protocols.

### 2.5. Statistical Analysis and Water Parameters Records

Statistical analysis in this study was performed using the Statistical Package for Social Sciences software, SPSS version 20. The multiple regression analysis was employed and in all cases, the significance level is set at 5% as discussed by Field [30]. The water parameters were measured in sea cage using Saltwater Master Test Kit, Aquarium Pharmaceuticals Index (API), USA. The procedure for each test was done according to manufacturer's instructions.

## 3. Results and Discussion

### 3.1. Morphological Analysis of* Neobenedenia melleni*

**Figure 1:**
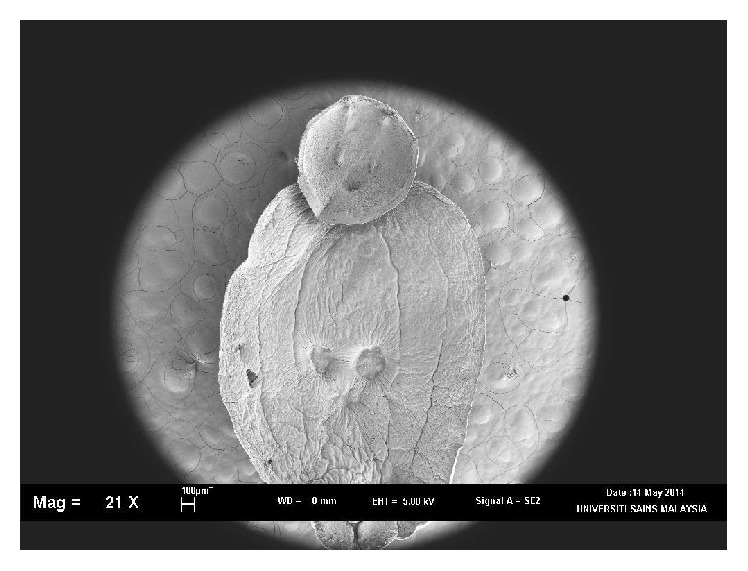
Ventral view of whole in SEM,* Neobenedenia melleni*.

**Figure 2 fig2:**
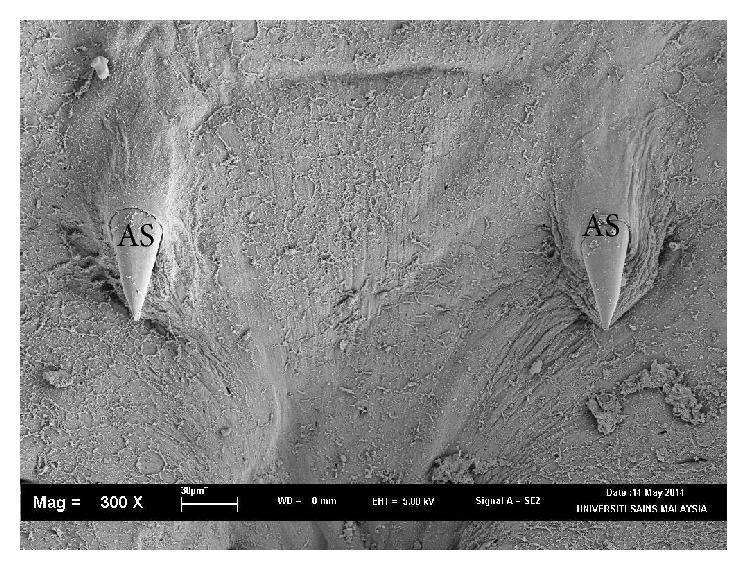
Ventral view of* Neobenedenia melleni* in SEM; AS: accessory sclerite; 40 *µ*m.

**Figure 3 fig3:**
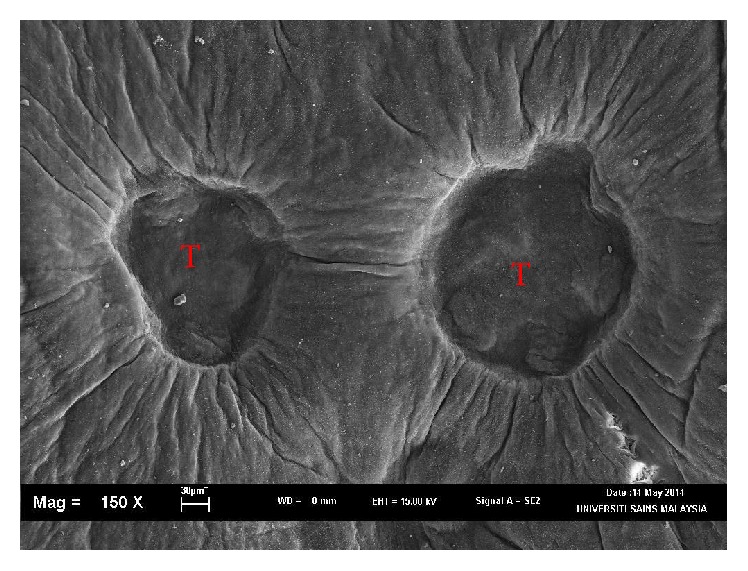
Ventral view of* Neobenedenia melleni* in SEM; T = testis organs.

**Figure 4 fig4:**
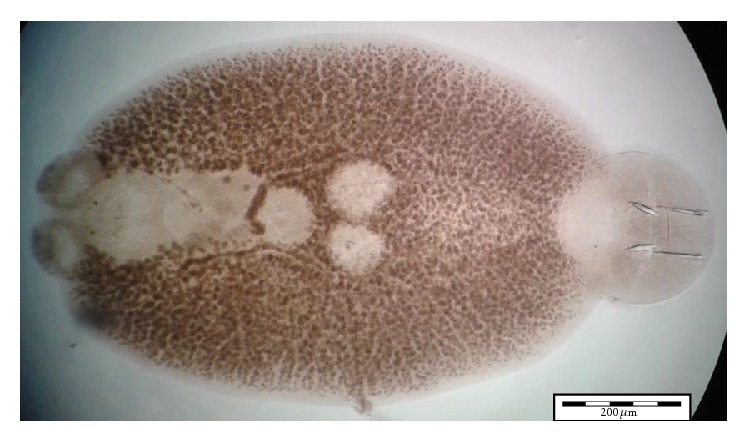
Ventral view of whole* Neobenedenia melleni* in compound microscope.

**Figure 5 fig5:**
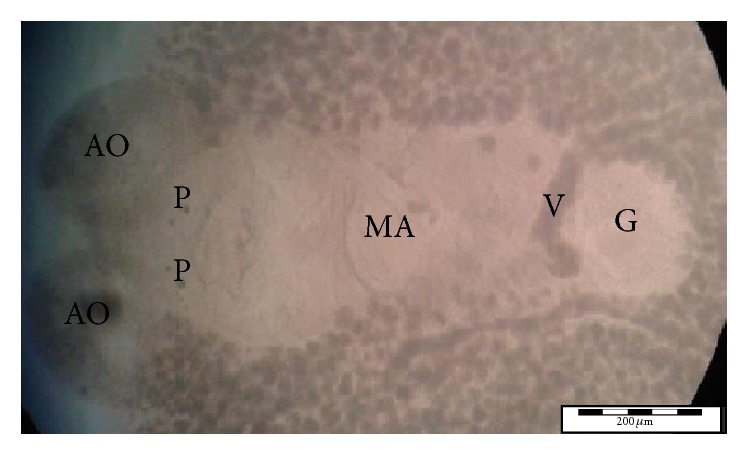
Ventral view of* Neobenedenia melleni* in compound microscope; AO: anterior attachment organ; P: pigmented eye; MA; male accessory gland reservoir; G: gland of Goto; V: vitelline reservoir.

**Figure 6:**
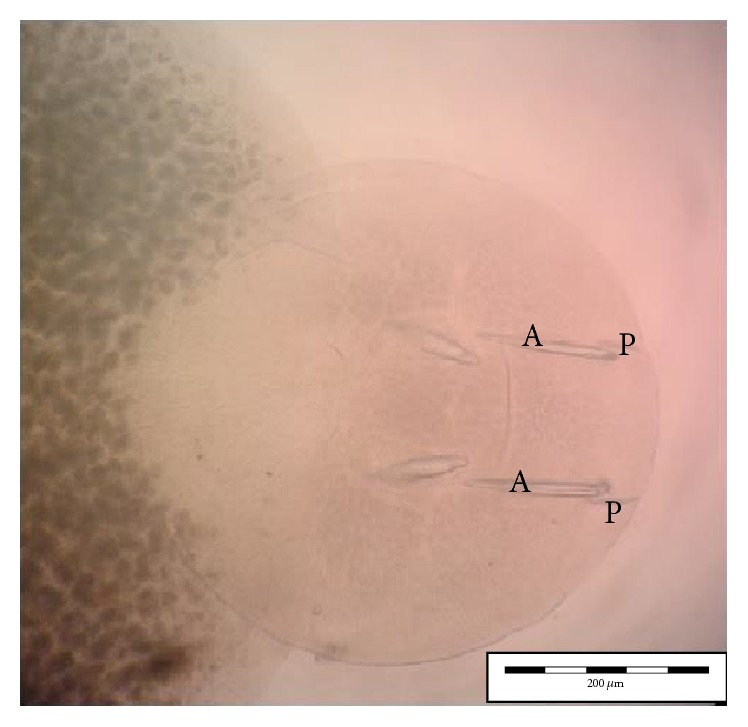
Ventral view of* Neobenedenia melleni* in compound microscope; A: anterior hamulus 150 *µ*m; P: posterior hamulus 40 *µ*m.

Using morphological key as described by Lawler [31] and Bullard et al. [32, 33]. In revising the generic diagnosis for* Neobenedenia*, Whittington and Horton [23] noted a variety of forms, which were more than 80 specimens attributed to* Neobenedenia melleni* from various host species. We are able to identify the parasite collected as* Neobenedenia melleni* according to Figures 1 [Fig fig2] [Fig fig3] [Fig fig4] [Fig fig5]–6, AS: accessory sclerite, 40 *μ*m; T: testis organs; AO: anterior attachment organ; P: pigmented eye; MA: male accessory gland reservoir; G: gland of Goto; V: vitelline reservoir; A: anterior hamulus, 150 *μ*m; P: posterior hamulus, 40 *μ*m. Total length of a sample specimen,* Neobenedenia melleni*, in this study, was recorded as 1050 *μ*m. The width length is recorded as 700 *μ*m.

### 3.2. DNA and Phylogenetic Analysis

**Figure 7:**
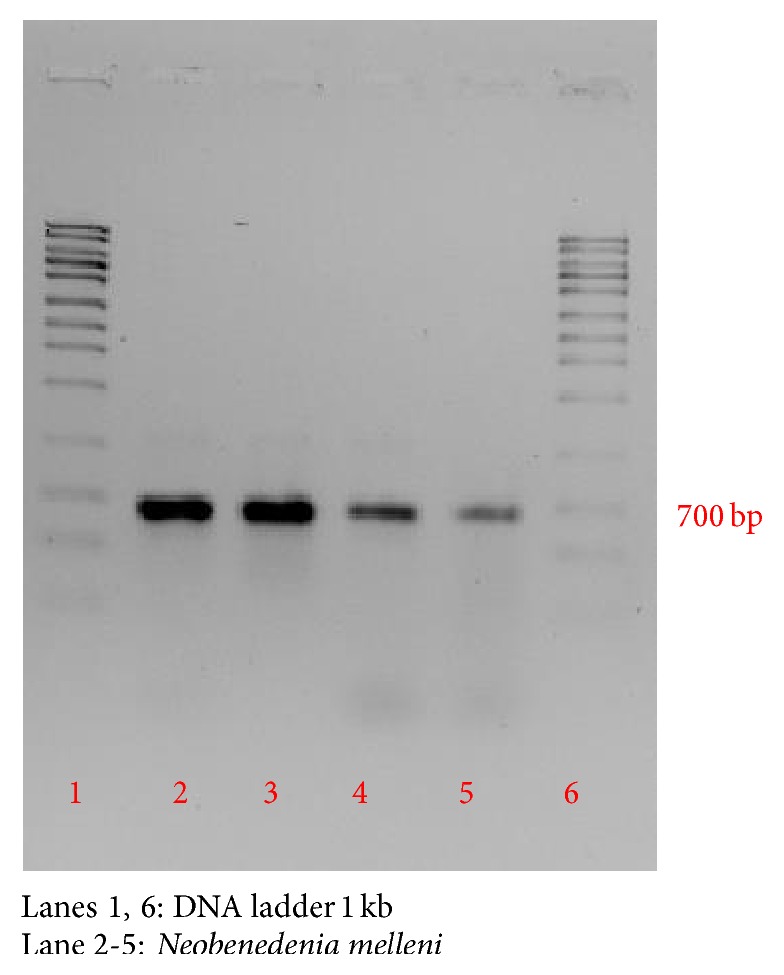
Gel electrophoresis of PCR 18s ribosomal RNA gene.

**Figure 8:**
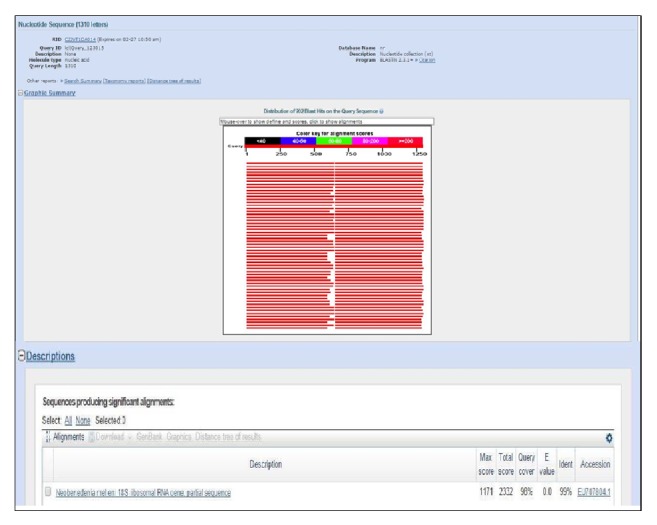
Nucleotide BLAST sequence for PCR product.

**Figure 9:**
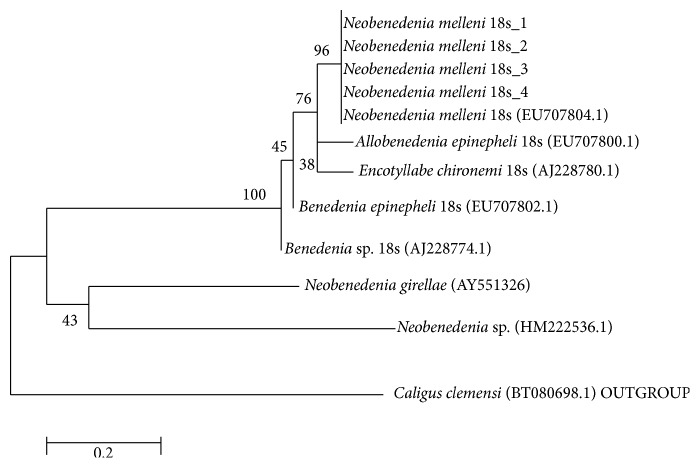
Constructed phylogenetic tree for* Neobenedenia melleni*.

Based on the results obtained upon gel electrophoresis analysis of the DNA template and PCR product, clearly visible bands were detected around 700 bp sequence, whereby this analysis was referred to Lucigen 1 kb DNA marker (USA) (Figure 7). Besides that, the optical density (OD) ratio of DNA was 2.0 with concentration of 135 (ng/*μ*L) for genomic DNA. The DNA sequence was further analyzed using Clustal W, Bioedit Software. The 18s sequence was successfully analyzed, recovered from all* Neobenedenia melleni* individuals. Nucleotide BLAST sequence for 18s ribosomal RNA gene from this study has shown 99% similarity with* N. melleni *EU707804.1 from GenBank dataset, as shown in Figure 8. Meanwhile, Figure 9 shows the constructed phylogenetic tree which shows two closely related clades between species.

All the individuals of* Neobenedenia melleni* recorded in this study showed a close relationship between species that was recorded from NCBI,* Neobenedenia melleni* EU707804.1, as 96% bootstrap value, followed by 76% of similarity between* Allobenedenia epinepheli* EU707800.1. The least similarity was recorded with* Encotyllabe chironemi* AJ228774.1,* Benedenia epinepheli* EU707802.1, and* Neobenedenia girellae* AY551326. Finally, all the sequence of* Neobenedenia melleni* in this study was deposited in GenBank with accession numbers KU843501, KU843502, KU843503, and KU843504. All this sequence is available as public database in GenBank.

### 3.3. Statistical Modeling for* Neobenedenia melleni*

A total of all 379 fishes were infected by* Neobenedenia melleni* parasite out of 400 examined fishes in natural sea culture cage environment.  Table 1 shows the descriptive statistics of dependent variable and predictor variables involved in this study. The average number of the* Neobenedenia melleni* parasite found in examination of fishes is approximately 25 with a standard deviation of 2.8. The mean value of fish length is 24.3 cm with a standard deviation of 3.7. Meanwhile, the binary coded variables, water temperature (0 = 32°C, 1 = 33°C) and salinity (0 = 32 ppt, 1 = 33 ppt), both have a higher percentage of low temperature (54.4%) and salinity (59.4%) compared to its counterpart.

Table 2 shows that the variables are positively correlated with one another and are significant at 1%. A large correlation of 0.92 is observed between fish length and prevalence of* Neobenedenia melleni* (increase in fish length will increase the prevalence of* Neobenedenia melleni* parasite) and 0.437 between water salinity and prevalence of* Neobenedenia melleni* parasite (increase in water salinity increases the prevalence of* Neobenedenia melleni* parasite). Finally, a moderate correlation of 0.3 is observed between water temperature and prevalence of* Neobenedenia melleni* parasite [30].

According to Table 3, the variance inflation factor (VIF) values are less than 10.0 or 2.0 and the tolerance statistics are above 0.2 [30]. The tolerance statistics is the reciprocal of VIF or 1/VIF. Multicollinearity issues are negated because the values met more than the requirement of VIF, variance inflation factor, and tolerance statistics.

Table 4 shows the multiple correlation coefficients *R*, the correlation among all the independent variables (temperature, fish length, and salinity), and the dependent variable which is at value 0.912. The *R*-Square value shows that all the predictors account for 84.5% of variation in the prevalence of parasite. The adjusted *R*-Square value (0.843) is similar to that of *R*-Square indicating that if these data were collected from the population rather than a sample it would have a similar result. Therefore, the result from this sample is generalized to the entire population of* Neobenedenia melleni* parasite infesting in fishes, as discussed in Field [30].

Table 5 shows that the model is a significant fit to the data, at less than 5%. Thus, the model is significantly improved to the ability to predict the dependent variable, prevalence of* Neobenedenia melleni* parasite infesting in fishes [34].

Table 6 shows the parameter estimates of multiple regression modeling. Among the variables that significantly predict the prevalence of* Neobenedenia melleni* parasite are fish length and water temperature, both significant at 1% and 5%; however salinity is not a significant predictor of* Neobenedenia melleni* in this analysis. Furthermore, the increase in one unit of fish length, holding other variables constant, increases the prevalence of parasite by approximately 1 (0.7≈1) unit. Also, increasing the temperature from 32 to 33 degrees Celsius increases the number of parasite by approximately 0.32 units, holding other variables constant. The 95% confidence interval (CI) conforms to the results obtained by observing the parameter estimate for fish length and temperature within the confidence interval and within a positive confidence interval bound.

The model can be rewritten as

(1) Estimated  number  of  Neobenedenia  melleni  parasites=8.2+0.7∗Fish  Length+0.32∗Water  Temperature.

**Figure 10:**
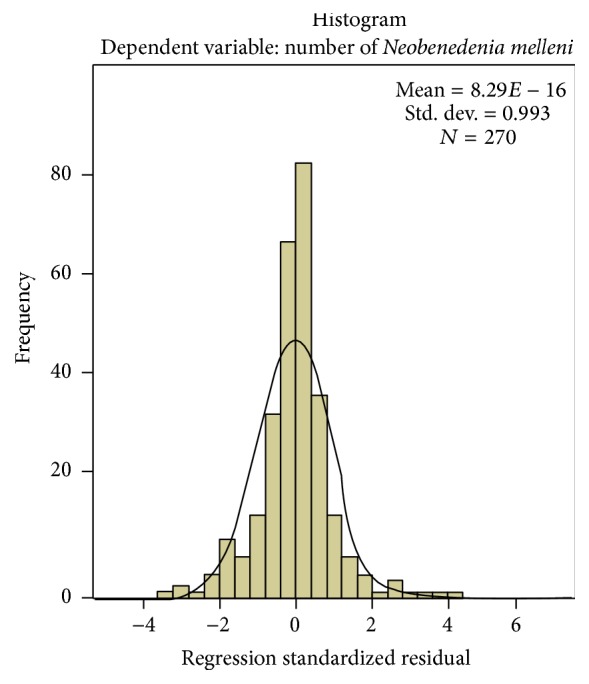
Histogram of the residuals data shows that the histogram has a bell shaped curve indicating that the residuals are normally distributed.

**Figure 11:**
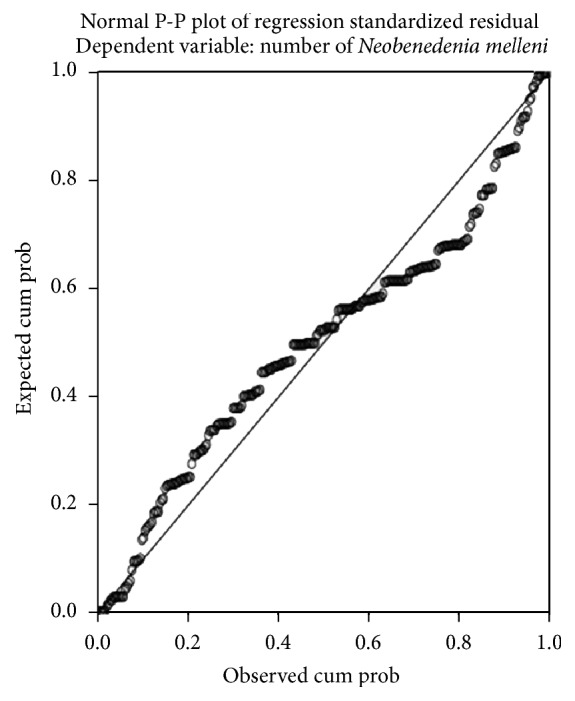
P-P plot shows that the points which represent the residuals lie along the diagonal line showing that the residuals are normally distributed.

**Figure 12:**
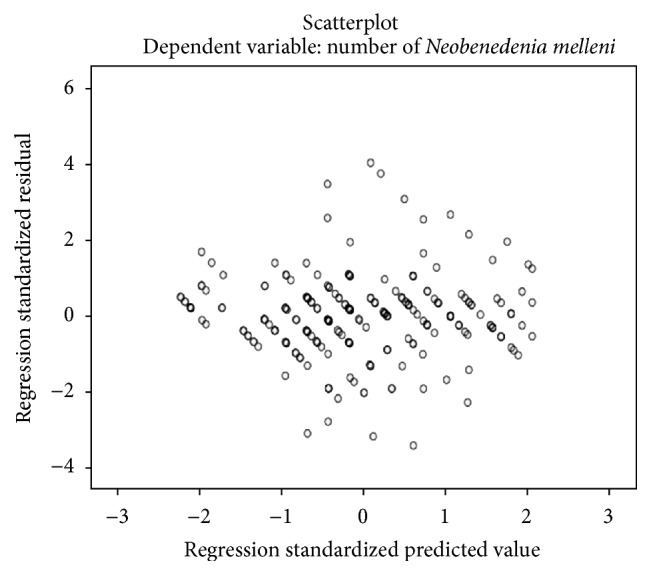
The figure shows that the points are randomly and evenly dispersed and do not have any specific pattern indicating that there is homoscedasticity or homogeneity of variance and linearity assumption is met.

Figure 10 shows the histogram of the residuals data which has a bell shaped curve indicating that the residuals are normally distributed. This is further verified by visualizing the normal P-P plot in Figure 11 which also shows that the points lie along a diagonal line indicating that the residuals are normally distributed.  Figure 12 shows that the points are randomly and evenly dispersed throughout the plot and concur with the assumptions of linearity and homoscedasticity of the residuals has been met as discussed in Field [30].

In this study, we have deployed multiple regression analysis method to observe biotic and abiotic factors that have influenced the miscellany of parasites in hosts, like fish length, water temperature, parasites count, and salinity [9]. These multiple variables are predicted to influence cultured fish and to come across rates with parasites and with the number of parasites that can endure in populations. A positive relationship is predicted among fish length, temperature, salinity, and parasite diversity because larger fish represent larger infection surface area for parasitic colonization [34, 35]. Besides that, temperature is mainly important as an environmental factor which merely controls the development period of parasitic copepods. Parasites, growth rates, egg production, survival rate, and conscription are reported to be high at higher water temperatures [21, 23]. The multiple regression analysis is integrated with the objective to produce a model that would best predict the optimal number of parasitic infestation based on observed values of three independent variables which were the length of fish, mean temperature, and salinity.

Several monogenean species exhibit short life cycles in warm temperatures [9, 23]. Accelerated parasitic life cycles will increase the metabolic and development rate associated with warm conditions [8, 14, 15]. Presently, the reason for the unpredictable and irregular nature of* Neobenedenia melleni* infection is unknown. Steps that can be implemented in reducing this rapid parasitic infestations are to have more attentive, frequent fish stock monitoring during warm, high temperature water conditions [36]. The major role of this temperature factors has been previously described by studies of life cycle of* Neobenedenia melleni*. Ogawa and Yokoyama [10] explained that* Neobenedenia melleni* took only 10 days to complete at 30°C as opposed to 20 days at 20°C in seawater.

Accordingly to Grau et al. [8], the hatching survival rate of* Neobenedenia melleni* eggs was less than 12% when incubated at salinity less than 18 ppt for 4 days.

## 4. Conclusion

In summary, this study has established an overview with statistical analysis for correlations of fish length and temperature that influences the number of fish parasites present in* Lutjanus erythropterus* fish species. Furthermore, morphology and DNA sequence identification were shown for* Neobenedenia melleni* parasite found in this cultured fish from Jerejak Island, Penang, Malaysia.

## Figures and Tables

**Table 1:** The table shows the descriptive statistics of dependent and predictor variables.

Characteristics	Frequency, *n*	Prevalence (%)	Mean	SD
Dependent variables				
*Neobenedenia melleni*	379	94.8^a^	25.24	2.8
Predictor variables				
Fish length	375	93.8^a^	24.3	3.7
Categorical variables				
Water temperature				
32.0	154	54.4		
33.0	129	45.6		
Water salinity				
32.0	168	59.4		
33.0	115	40.6		

*Note.* ^a^Total percentage is not 100% due to missing values; SD: standard deviation.

**Table 2:** Correlations between variables using bivariate analysis.

Variables	Prevalence of *Neobenedenia melleni*	Fish length	Water temperature	Water salinity
Prevalence of *Neobenedenia melleni*	**1**			
Fish length	0.917^*∗∗∗*^	**1**		
Water temperature	0.298^*∗∗∗*^	0.270^*∗∗∗*^	**1**	
Water salinity	0.437^*∗∗∗*^	0.428^*∗∗∗*^	0.384^*∗∗∗*^	**1**

Note: ^*∗∗∗*^significant at 1%.

**Table 3:** Variance inflation factor values of predictor variables.

Variables	Tolerance statistics	Variance inflation factor (VIF)
Fish length	0.751	1.332
Temperature	0.778	1.286
Salinity	0.659	1.518

**Table 4:** The model summary.

Multiple correlation coefficient, *R*	*R*-Square	Adjusted *R*-Square	Standard error of the estimate
0.912	0.845	0.843	1.124

**Table 5:** The model fit values.

	Sum of squares	df	Mean square	*F*-statistics	*P* value
Regression	1829.444	4	457.361	361.868	0.000
Residual	334.930	265	1.264		
Total	2164.374	269			

**Table 6:** Fitted values of the predictor variables via multiple regression analysis.

Variables	Parameter estimates, *β*	Standard error, (SE)	95% Confidence interval (CI)	
			Lower bound	Upper bound
Constant	8.244	0.465	7.329	9.159
Fish length	0.669^*∗∗∗*^	0.021	0.628	0.710
Temperature	0.319^*∗∗*^	0.156	0.013	0.625
Salinity	0.124	0.172	−0.214	0.462

*Note.* Significant at ^*∗∗∗*^1% and ^*∗∗*^5% significance level.

## References

[1] Chong Y. C., Chao T. M. (1984). Quarantine treatment of imported Epinephelus tauvina fry. *Singapore Veterinary Journal*.

[2] Chong Y. C., Chao T. M. (2011). *Common Diseases of Marine Food Fish*.

[3] Leong T. S. (1994). *Parasites and Diseases of Cultured Marine Finfishes in Southern Asia*.

[4] Leong T. S., Wong S. Y. (1989). Parasites of wild and cultured golden snapper, *Lutjanus johni* (Bloch) in Malaysia. *Tropical Biomedicine*.

[5] Leong T. S., Wong S. Y., Langdon J. S., Enriquez G. L., Sukimin S. (1992). Parasites of marine finfishes cultured in ponds and cages in Indonesia. *Tropical Fish Health Management in Aquaculture*.

[6] Leong T.-S., Wong S.-Y. (1988). A comparative study of the parasite fauna of wild and cultured grouper (Epinephelus malabaricus Bloch & Schneider) in Malaysia. *Aquaculture*.

[7] Leong T. S., Wong S. Y. (1990). Parasites of healthy and diseased juvenile grouper (*Epinephelus malabaricus* Bloch & *Schneider*) and seabass (*Lates calcarifer* Bloch) in floating cages in Penang, Malaysia. *Asian Fisheries Science*.

[8] Grau A., Crespo S., Pastor E., González P., Carbonell E. (2003). High infection by *Zeuxapta seriolae* (Monogenea: Heteraxinidae) associated with mass mortalities of amberjack Seriola dumerili Risso reared in sea cages in the Balearic Islands (Western Mediterranean). *Bulletin of the European Association of Fish Pathologists*.

[9] Tubbs L. A., Poortenaar C. W., Sewell M. A., Diggles B. K. (2005). Effects of temperature on fecundity in vitro, egg hatching and reproductive development of *Benedenia seriolae* and *Zeuxapta seriolae* (Monogenea) parasitic on yellowtail kingfish *Seriola lalandi*. *International Journal for Parasitology*.

[10] Ogawa K., Yokoyama H. (1998). Parasitic diseases of cultured marine fish in Japan. *Fish Pathology*.

[11] Littlewood D. T. J., Rohde K., Bray R. A., Herniou E. A. (1999). Phylogeny of the Platyhelminthes and the evolution of parasitism. *Biological Journal of the Linnean Society*.

[12] Boeger W. A., Kritsky D. C., Littlewood D. T. J., Bray R. A. (2001). Phylogenetic relationships of the Monogenoidea. *Interrelationships of the Platyhelminthes*.

[13] Lockyer A. E., Olson P. D., Littlewood D. T. J. (2003). Utility of complete large and small subunit rRNA genes in resolving the phylogeny of the Neodermata (Platyhelminthes): implications and a review of the cercomer theory. *Biological Journal of the Linnean Society*.

[14] Bychowsky B. E. *Monogenetic Trematodes, Their Systematics and Phylogeny*.

[15] Justine J.-L. (1991). Cladistic study in the Monogenea (Platyhelminthes), based upon a parsimony analysis of spermiogenetic and spermatozoal ultrastructural characters. *International Journal for Parasitology*.

[16] Justine J.-L. (1998). Non-monophyly of the monogeneans?. *International Journal for Parasitology*.

[17] Llewellyn J. (1970). Taxonomy, genetics and evolution of parasites. *The Journal of Parasitology*.

[18] Mollaret I., Jamieson B. G. M., Justine J.-L. (2000). Phylogeny of the Monopisthocotylea and Polyopisthocotylea (Platyhelminthes) inferred from 28S rDNA sequences. *International Journal for Parasitology*.

[19] Littlewood D. T. J., Bray R. A., Clough K. A. (1998). A phylogeny of the Platyhelminthes: towards a total-evidence solution. *Hydrobiologia*.

[20] Leong T. S., Wong S. Y. (1995). Parasites of grouper, *Epinephelus suillus* from Pulau Langkawi and Kelantan, Malaysia. *Journal Bioscience*.

[21] Yamaguti S. (1963). *Systema Helminthum, Volume IV: Monogenea and Aspidocotylea*.

[22] Li A.-X., Wu X.-Y., Ding X.-J. (2005). PCR-SSCP as a molecular tool for the identification of *Benedeniinae* (Monogenea: Capsalidae) from marine fish. *Molecular and Cellular Probes*.

[23] Whittington I. D., Horton M. A. (1963). A revision of *Neobenedenia* Yamaguti, 1963 (Monogenea: Capsalidae) including a redescription of *N. melleni* (MacCallum, 1927) Yamaguti. *Journal of Natural History*.

[24] Kabata Z. (1979). *Parasitic Copepoda of British Fishes*.

[25] Kua B. C., Abdullah S. Z., Abtholuddin M. F., Mohd N. F., Mansor N. N. Marine leech isolated from cage-cultured sea bass (Lates calcarifer) fingerlings: a parasite or Vector.

[26] Kua B. C., Faizul H. (2010). Scanning electron microscopy of three species of *Caligus* (Copepoda:Caligidae) parasitized on cultured marine fish at Bukit Tambun, Penang. *Malaysian Journal of Microscopy*.

[27] Dang B. T., Levsen A., Schander C., Bristow G. A. (2010). Some *Haliotrema* (Monogenea: Dactylogyridae) from cultured grouper (*Epinephelus* Spp.) with emphasis on the phylogenetic position of *Haliotrema cromileptis*. *Journal of Parasitology*.

[28] Tamura K., Dudley J., Nei M., Kumar S. (2007). MEGA4: Molecular Evolutionary Genetics Analysis (MEGA) software version 4.0. *Molecular Biology and Evolution*.

[29] Kimura (1984). Estimation of evolutionary distance between nucleotide sequences. *Molecular Biology and Evolution*.

[30] Field A. (2005). *Discovering Statistics Using SPSS*.

[31] Lawler A. R. (1981). *Zoogeography and Host-Specificity of the Superfamily Capsaloidea Price, 1936 (Monogenea: Monopisthocotylea). An Evaluation of the Host-Parasite Locality Records of the Superfamily Capsaloidea Price, 1936, and Their Utility in Determinations of Host-Specificity and Zoogeography*.

[32] Bullard S. A., Benz G. W., Braswell J. S. (2000). *Dionchus* postoncomiracidia (Monogenea: Dionchidae) from the skin of blacktip sharks, *Carcharhinus limbatus* (Carcharhinidae). *Journal of Parasitology*.

[33] Bullard S. A., Benz G. W., Overstreet R. M., Williams E. H., Hemdal J. (2000). Six new host records and an updated list of wild hosts for *Neobenedenia melleni* (MacCallum) (Monogenea: Capsalidae). *Comparative Parasitology*.

[34] Ravi R., Yahaya Z. S. (2015). Relationship between size of fish, temperature and parasitic intensity in snakehead fish species from Kepala Batas, Penang, Peninsular Malaysia. *Pertanika Journal of Tropical Agricultural Science*.

[35] Ravi R., Yahaya Z. S. DNA barcoding of marine leeches (*Zeylanicobdella argumensis*) from crimson red snapper (*Lutjanus eryhtopterus*), peninsular Malaysia and their phylogenetic analysis.

[36] Main K. L., Rosenfeld C. (1995). *Culture of Highvalue Marine Fishes in Asia and the United States*.

